# Na_V_1.6 and Na_V_1.7 channels are major endogenous voltage-gated sodium channels in ND7/23 cells

**DOI:** 10.1371/journal.pone.0221156

**Published:** 2019-08-16

**Authors:** Jisoo Lee, Shinae Kim, Hye-mi Kim, Hyun Jeong Kim, Frank H. Yu

**Affiliations:** 1 Department of Pharmacology and Dental Therapeutics, Program in Neurobiology, Dental Research Institute, Seoul National University School of Dentistry, Seoul, Republic of Korea; 2 Department of Dental Anesthesiology, Program in Neurobiology, Dental Research Institute, Seoul National University School of Dentistry, Seoul, Republic of Korea; Cooper Medical School of Rowan University, UNITED STATES

## Abstract

ND7/23 cells are gaining traction as a host model to express peripheral sodium channels such as Na_V_1.8 and Na_V_1.9 that have been difficult to express in widely utilized heterologous cells, like CHO and HEK293. Use of ND7/23 as a model cell to characterize the properties of sodium channels requires clear understanding of the endogenous ion channels. To define the nature of the background sodium currents in ND7/23 cells, we aimed to comprehensively profile the voltage-gated sodium channel subunits by endpoint and quantitative reverse transcription-PCR and by whole-cell patch clamp electrophysiology. We found that untransfected ND7/23 cells express endogenous peak sodium currents that average –2.12nA (n = 15) and with kinetics typical of fast sodium currents having activation and inactivation completed within few milliseconds. Furthermore, sodium currents were reduced to virtually nil upon exposure to 100nM tetrodotoxin, indicating that ND7/23 cells have essentially null background for tetrodotoxin-resistant (TTX-R) currents. qRT-PCR profiling indicated a major expression of TTX-sensitive (TTX-S) Na_V_1.6 and Na_V_1.7 at similar levels and very low expression of TTX-R Na_V_1.9 transcripts. There was no expression of TTX-R Na_V_1.8 in ND7/23 cells. There was low expression of Na_V_1.1, Na_V_1.2, Na_V_1.3 and no expression of cardiac or skeletal muscle sodium channels. As for the sodium channel auxiliary subunits, β1 and β3 subunits were expressed, but not the β2 and β4 subunits that covalently associate with the α-subunits. In addition, our results also showed that only the mouse forms of Na_V_1.6, Na_V_1.7 and Na_V_1.9 sodium channels were expressed in ND7/23 cells that was originally generated as a hybridoma of rat embryonic DRG and mouse neuroblastoma cell-line. By molecular profiling of auxiliary β- and principal α-subunits of the voltage gated sodium channel complex, our results define the background sodium channels expressed in ND7/23 cells, and confirm their utility for detailed functional studies of emerging pain channelopathies ascribed to mutations of the TTX-R sodium channels of sensory neurons.

## Introduction

Voltage-gated sodium channels (VGSC) are responsible for initiation and propagation of the action potential in central and peripheral neurons and most excitable cells such as heart and skeletal muscle [1]. Sodium channels likely function as a multimeric complex as many of these pore-forming principal α-subunits associate natively with auxiliary subunits, β1 to β4, that modulate sodium channel gating, regulate channel density, as well as have cell adhesion functions independent of the α-subunits [1–3]. The VGSC family consist of nine pore-forming α-subunits (Na_V_1.1 to Na_V_1.9) and can be distinguished into two groups by their relative susceptibility for tetrodotoxin (TTX) blockade, a neurotoxin found in puffer fish. TTX-resistant (TTX-R) Na_V_1.5, Na_V_1.8 and Na_V_1.9 channels are little affected by and therefore ‘resistant’ to nanomolar concentrations of the TTX that fully block other sodium channel isoforms highly expressed in the central nervous system or skeletal muscle (Na_V_1.1, Na_V_1.2, Na_V_1.3, Na_V_1.4, Na_V_1.6, Na_V_1.7). The IC_50_ for the TTX-R sodium channels are in the micromolar range which is approximately 1000-fold greater than the TTX-sensitive (TTX-S) channels [4].

The Na_V_1.8 and Na_V_1.9 channels show preferential expression in the peripheral sensory nerves, especially in the dorsal root ganglia (DRG) and trigeminal ganglia, and play critical roles in nociception and pain pathways [5]. These channels are important drug discovery targets for pain [6], yet our understanding of these channels have been limited because unlike many TTX-S sodium channels, recombinant Na_V_1.8 (TTX-R) is poorly expressed in many heterologous systems. They have reduced transfection rates in COS-7 cells [7], low current levels in CHO cells that were not consistently repeatable [8], loss of expression upon thawing of passaged HEK293 stable cell-lines [9], as well as abnormally long induction time to attain low expression in *Xenopus* oocytes [10]. Furthermore, sodium currents that were recorded following Na_V_1.8 cDNA transfection have markedly depolarized current-voltage relationship and slower inactivation kinetics compared with endogenous TTX-R currents in DRG [7]. Similarly, recombinant TTX-R Na_V_1.9 channels have also been historically difficult to express in mammalian cell for reasons that are not clear. Individual gene knockout neurons of Na_V_1.8 and Na_V_1.9 also exist [11, 12]; however, detailed structure/function or pharmacological studies using acutely isolated neurons are less efficient than cell lines, requiring much time and resources. Attempts to generate transient or stably-transfected HEK293 and CHO cells have also been difficult [13], although breakthrough stable expression of recombinant Na_V_1.9 in HEK293 was attained from a pharmaceutical industry research group recently [14].

The ND7/23 is a fusion cell-line of embryonic rat DRG and mouse N18Tg2 neuroblastoma, established to model nociceptive sensory neuron-like properties [15]. It has been used recently to express recombinant TTX-R sodium channels, specifically the peripheral sodium channels, Na_V_1.8 and Na_V_1.9 [16]. Moreover, stably expressing the rat Na_V_1.8 cDNA in these cells yielded biophysical properties that closely resembled those of the native rat DRG TTX-R sodium currents [9]. Vanoye *et al*. (2013) reported stable expression of human Na_V_1.9 using high-efficiency transposon system and low-temperature incubation, after unsatisfactory trials to generate stably transfected HEK293 and CHO cell lines [17]. The heterologous expression of Na_V_1.9 channels in these cells suggest that differential expression of factors present in neuron-derived ND7/23 cells compared to the epithelia-derived cells [17] may encumber the expression of TTX-R sodium channels in widely-used cells such as HEK293.

In order to transfect and to characterize properties of recombinant sodium currents in the ND7/23 cell model, the levels and identities of endogenous sodium channels expressed in these cells must be understood. To define the nature of the background sodium currents in ND7/23 cells, we profiled the VGSC expression in ND7/23 cells by reverse transcription-PCR. We report that ND7/23 cells contain Na_V_1.7 and Na_V_1.6 transcripts that comprise the main TTX-S sodium currents. There was no Na_V_1.8 channels and very low expression of mouse TTX-R Na_V_1.9 transcripts as well as TTX-S Na_V_1.1, Na_V_1.2, Na_V_1.3. In addition, auxiliary β1 and β3 subunits of sodium channels were expressed in ND7/23 cells, but not the β2 nor β4 subunits that covalently associate with sodium channel α-subunits.

## Results

### Properties of sodium current in ND7/23 cells

**Fig 1:**
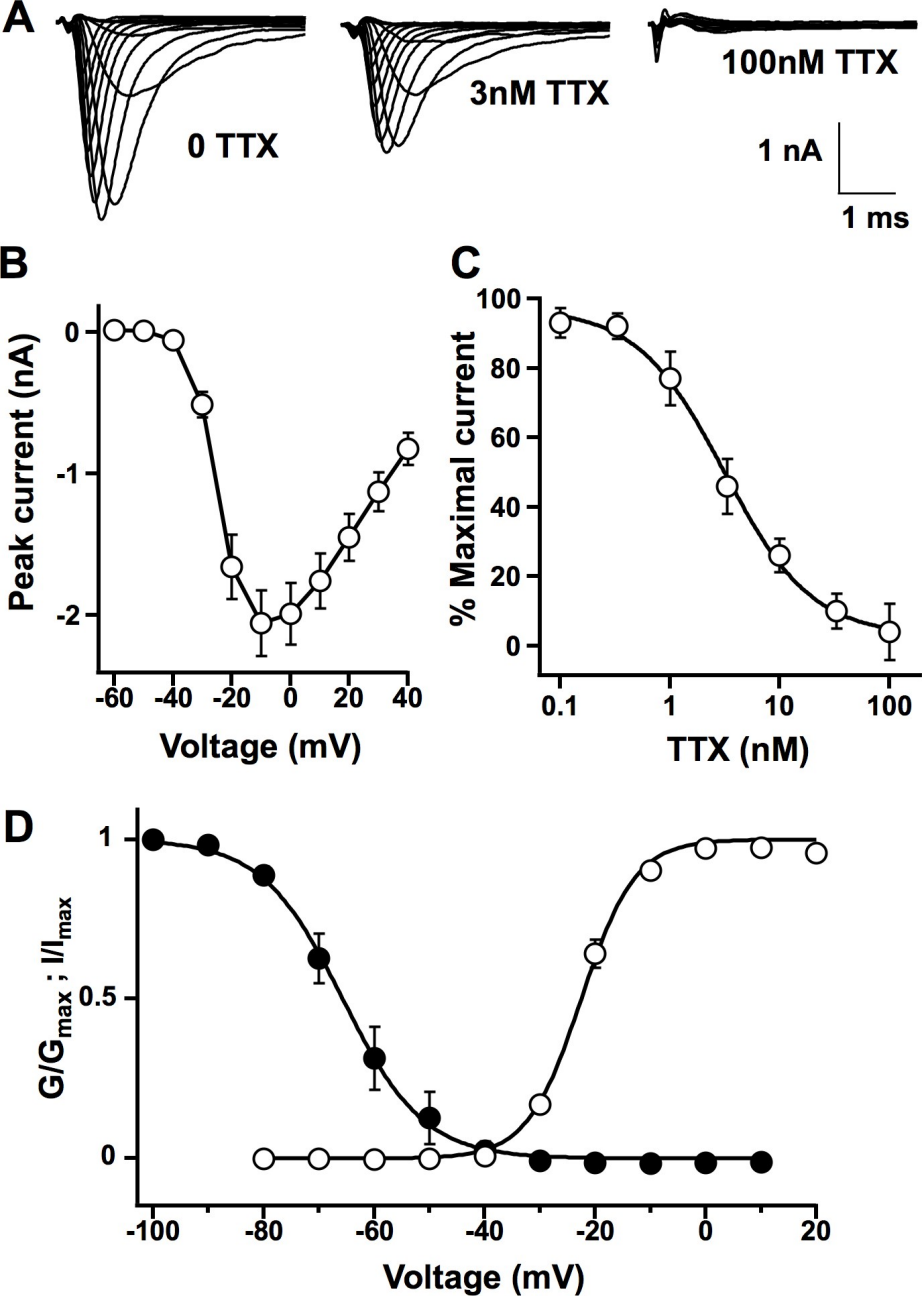
Endogenous sodium currents of ND7/23 cells. (A) Families of sodium current traces recorded from untransfected ND7/23 cells under perfusion of external solution without (*left*) and with 3nM (*middle*) and 100nM (*right*) TTX. Calibration: 1nA, 1ms. (B) Current-voltage relation of the sodium current elicited by 100 ms square test pulse from –80 to +50mV in 10mV steps preceded by –120mV prepulse for 150ms and -70mV holding potential (means±standard error, *n* = 15). (C) TTX inhibition curve for the sodium current. Each data point was acquired using step depolarization to 0mV from –120mV and normalized to peak sodium current elicited under control perfusion prior to TTX application (means±SD, *n* = 3~11). IC_50_ was 3.3±1.0nM. (D) Conductance–voltage (open circle) and voltage-dependence of the steady-state inactivation (filled circle) relationships of ND7/23 cell sodium currents. Steady state inactivation used test pulse to 0mV, preceded by 500ms long prepulses to the indicated potentials and the holding potential was –120mV.

By whole-cell patch clamp electrophysiology, robust inward peak sodium currents of –2.1±0.9nA (SE, Fig 1A) were recorded endogenously in these cells similar to prior studies [9, 18, 19] The activation and inactivation of the sodium currents were complete within ~2 milliseconds, consistent with the fast kinetics found in many TTX-sensitive (TTX-S) sodium channels. Bath perfusion of TTX produced concentration-dependent block of the sodium currents with approximately half-block by 3nM and complete block by 100nM (Fig 1A). Current-voltage relationship shows that the endogenous sodium currents activate positive to -40mV and peak near -10mV (Fig 1B). The IC_50_ of the TTX inhibition in ND7/23 cells was 3.3±1.0nM (Fig 1C) which is typical of TTX-S sodium currents with IC_50_ values in the single-digit nanomolar range [4]. The voltage dependence of activation and steady-state inactivation is presented in Fig 1D. It showed midpoint of activation at –22.5±0.6mV (*n* = 7) with slope of 4.9 and midpoint of steady-state inactivation at –65.7±0.3mV (*n* = 7) with slope of –7.3. Overall, the kinetics, the voltage-dependence, and the sensitivity to the TTX-block of the sodium currents in ND7/23 cells as presented in Fig 1 are consistent with those of neuronal TTX-S sodium channels.

### Expression of voltage-gated sodium channel isoforms in ND7/23 cells

**Fig 2:**
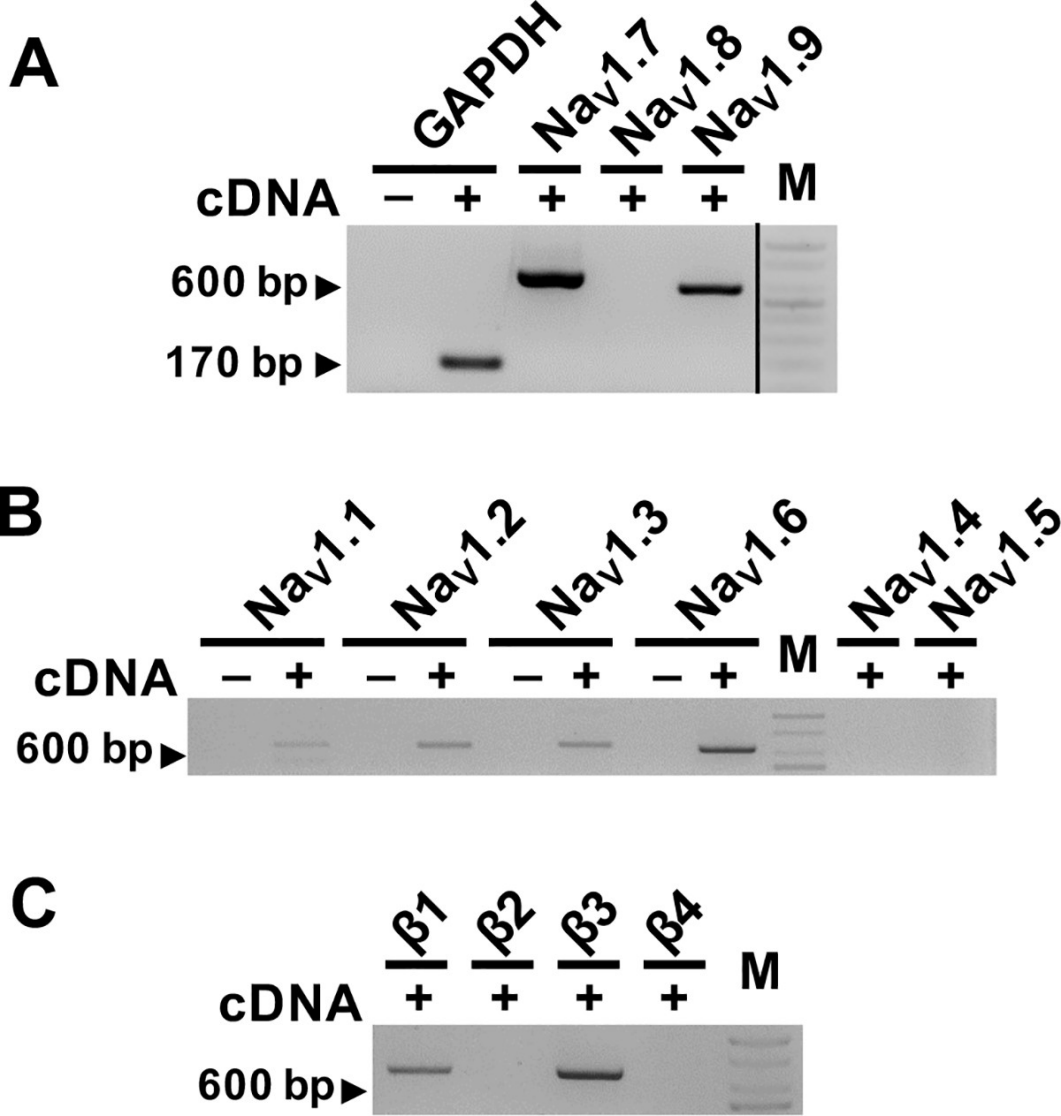
RT-PCR analysis of voltage gated sodium channel isoforms in ND7/23 cells. (A) Expression of Na_V_1.7, Na_V_1.8, Na_V_1.9 sodium channel α-subunits in ND7/23 transcriptome. DNA marker run on non-adjacent lane on same gel (S2 Fig). Expected amplicon sizes were respectively (in bp), 656, 521, and 564. The + and–indicate presence and absence of cDNA in PCR reaction. Gapdh, Glyceraldehyde 3-phosphate dehydrogenase; M, DNA size marker (100 bp). (B), Expression of Na_V_1.1, Na_V_1.2, Na_V_1.3, Na_V_1.4, Na_V_1.5, Na_V_1.6 sodium channel α-subunits. Expected amplicon sizes were respectively (in bp), 683, 674, 681, 368, 589, and 662. (C) Expression of sodium channel auxiliary subunits β1, β2, β3, β4, in ND7/23 transcriptome. Expected amplicon sizes were respectively (in bp), 657, 648, 648, and 687. Gels are representative of at least three independent experiments.

Molecular characterization of the sodium channels expressed natively in ND7/23 cells were done by RT-PCR screen to amplify the transcripts of the sodium channel isoforms. The cDNA was reverse-transcribed from ND7/23 cell homogenates using oligo dT. We first screened for the sodium channel isoforms that are highly expressed in the peripheral nervous system with gene-specific primers (S1 Table). There was a robust amplification of Na_V_1.7 transcripts, weak amplification of Na_V_1.9 and no signal for Na_V_1.8 transcripts (Fig 2A). Although it was not quantified, PCR amplification of Na_V_1.7 appeared to be at a level similar to that of Gapdh control, suggesting that Na_V_1.7 may comprise a large portion of the endogenous sodium currents in ND7/23 cells. As transcripts of other sodium channel isoforms are present natively in DRG [5], we investigated the expression of Na_V_1.1, Na_V_1.2, Na_V_1.3 and Na_V_1.6 (central nervous system) which showed mostly weak but distinct PCR bands (Fig 2B). Among these, Na_V_1.6 showed the strongest expression which appeared to be consistently similar to Na_V_1.7 levels (S1 Fig). There was no PCR amplification when Na_V_1.4-specific (skeletal) or Na_V_1.5-specific (cardiac) primer sets were used. A third PCR screen of ND7/23 mRNA was performed to characterize the expression of auxiliary β subunits (β1 to β4) of the voltage-gated sodium channel complex. As presented in Fig 2C, it showed that only the β1 and β3 subunits PCR bands were amplified, but β2 and β4 were not, similar to the native expression in rat DRG [20], and confirms prior study in ND7/23 cells by John *et al*. (2004) [9].

**Fig 3:**
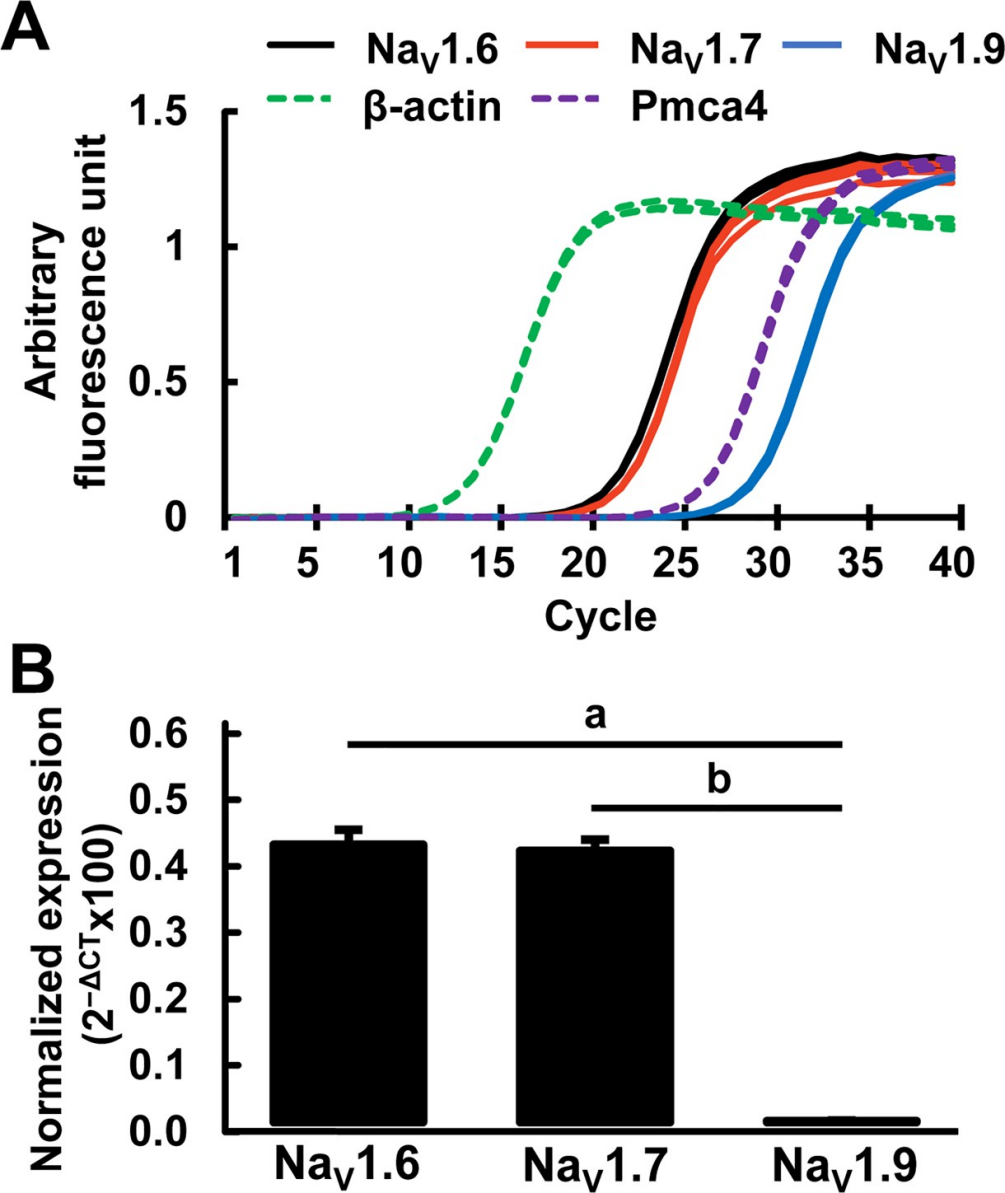
Quantitative RT-PCR analysis of Na_V_1.6, Na_V_1.7, Na_V_1.9 sodium channel transcripts in ND7/23 cells. (A) Real-time change in fluorescence with PCR cycle number in a representative of qRT-PCR experiment. Each primer set was determined in technical triplicates and data are plotted as lines. β-actin and Pmca4 (Plasma membrane calcium ATPase4) were controls. (B) Relative expression of Na_V_1.6, Na_V_1.7, Na_V_1.9 transcript averaged from three independent experiments (total RNA samples) and each data point (*ie*, primer set) was averaged from technical triplicates and normalized to reference gene (β-actin). Mean±SD (n = 3); Comparisons by one way ANOVA followed by post-hoc Student’s *t*-test with Bonferroni correction. a,b, *p* < 0.001.

Consistent with earlier studies that have amplified Na_V_1.2 and Na_V_1.3 in ND7/23 [21], we also detected Na_V_1.2, Na_V_1.3, and Na_V_1.1 PCR bands *albeit* at lower levels than Na_V_1.7 (S1 Fig). To compare the relative expression of transcripts, we selected Na_V_1.6, Na_V_1.7, and Na_V_1.9 for quantitative analysis by real-time PCR (qRT-PCR). Three independent total RNA samples of ND7/23 cells were analyzed by qRT-PCR experiments using each primer set in triplicate. A plot of the data from one representative qRT-PCR experiment is presented in Fig 3A. Expression level of the sodium channels were many orders of magnitude lower than the β-actin control. Plasma membrane calcium ATPase4 (Pmca4) expressed in nervous system was used as a quality control indicator (plasma membrane transport protein housekeeping gene) between qRT-PCR experiments [22]. The levels of Na_V_1.6 and Na_V_1.7 transcripts were similar and much higher than Pmca4, and Na_V_1.9 showed the lowest level of expression. Compared to the mRNA expression of Na_V_1.6 and Na_V_1.7, the expression of Na_V_1.9 was approximately 270-fold lower, consistent with lack of TTX-R sodium currents recorded in the presence of 100nM TTX in ND7/23 cells (Fig 1).

### Na_V_1.8 is not expressed in ND7/23 cells

**Fig 4:**
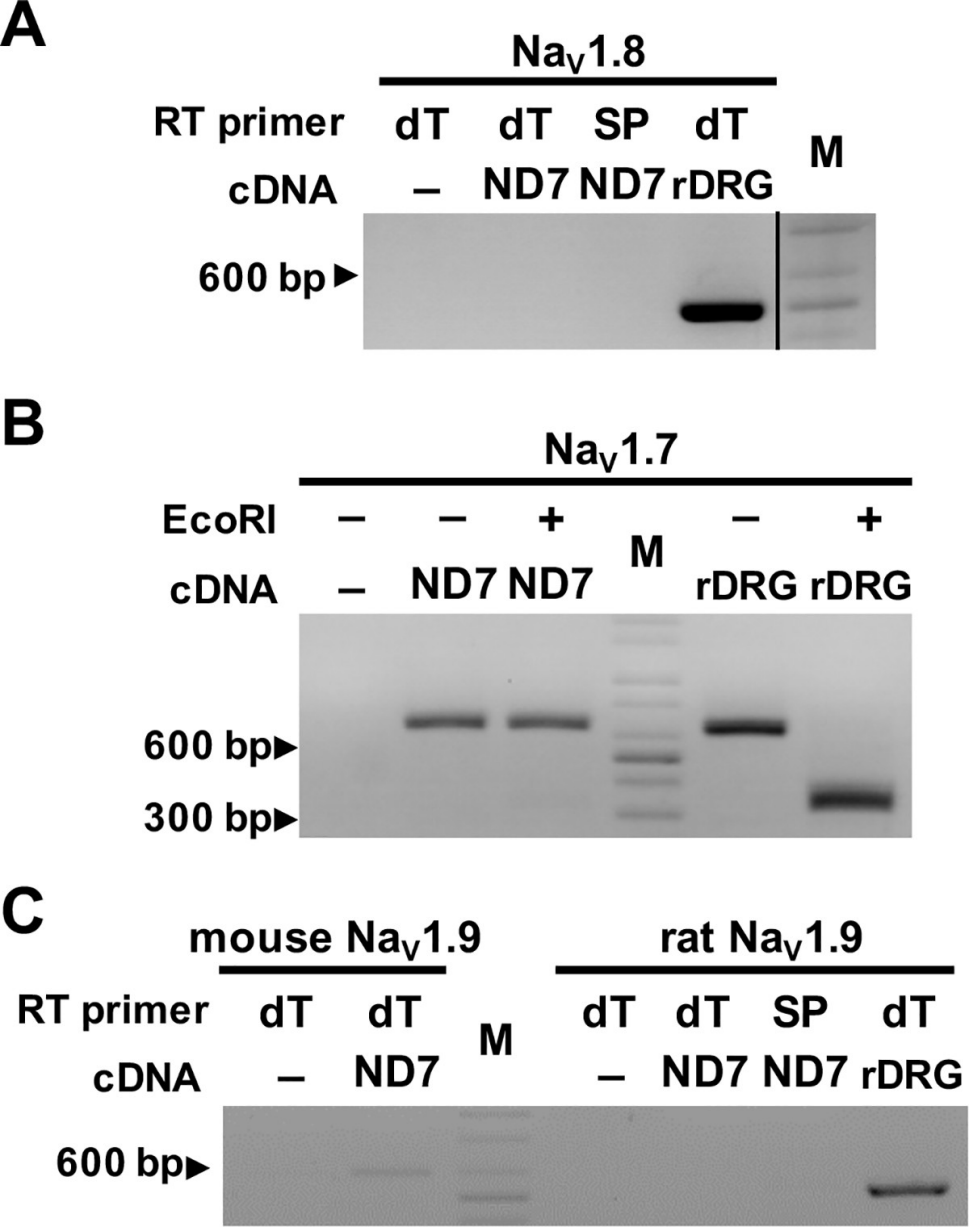
Genomic origin of sodium channels in ND7/23 cells. (A) RT-PCR of Na_V_1.8 from ND7/23 cDNA synthesized with oligo-dT or Na_V_1.8-specific (SP) reverse primer. DNA marker run on non-adjacent lane on same gel (S2 Fig). Expected amplicon size in mouse or rat is 521 bp. The–symbol indicate absence of cDNA in PCR reaction. ND7, ND7/23; rDRG, rat dorsal root ganglion; M, DNA size marker (100 bp). (B) *Eco*RI restriction enzyme cleavage of Na_V_1.7 amplicons from ND7/23 cells. Mouse Na_V_1.7 sequence does not contain the *Eco*RI restriction site that rat Nav1.7 sequence harbors within. The–and + symbols indicate absence or addition of *Eco*RI enzyme to Na_V_1.7 amplicons. (C) RT-PCR of ND7/23 cDNA synthesized with oligo-dT or Na_V_1.9-specific (SP) reverse primers using mouse-Nav1.9-b primer set (606 bp, different primers from that used in Fig 2A) and rat-specific Na_V_1.9 primer set. Gels are representative of at least three independent experiments.

Our initial screen of sodium channel isoforms using oligo-dT primed cDNA showed no signal for Na_V_1.8. Reverse transcription is sensitive to secondary RNA structures found in many 3’-untranslated regions of mRNAs and differences in priming strategies used in the cDNA synthesis can lead to large variations in relative gene expression measurements [19]. Additional RT-PCR experiment was performed using a reverse primer to the coding region of Na_V_1.8 channel for the cDNA synthesis. It showed that Na_V_1.8-specific band was not amplified from ND7/23 cell whereas the same primer set whose sequences are also identical to rat Na_V_1.8, produced an intense RT-PCR amplification of Na_V_1.8 from the rat DRG (Fig 4A). The Na_V_1.8 primer set used in Figs 4A and 2A produced expected 521 bp from a full-length mouse Na_V_1.8 cDNA as part of our routine quality control PCR to test primer design (S1 Fig), suggesting that lack of Na_V_1.8 signal in ND7/23 cells is not due to failure to recognize mouse sequence. Overall, these results confirm that Na_V_1.8 channels are not expressed in ND7/23 cells.

### VGSCs in ND7/23 cells are mouse isoforms

ND7/23 cell is a hybrid cell line generated by fusion of mouse neuroblastoma and rat DRG [15]; however, the genomic origin of the voltage-gated sodium channels in ND7/23 cells is unclear. We designed additional experiments to discriminate whether the Na_V_1.6, Na_V_1.7 and Na_V_1.9 expressed in ND7/23 cell are from mouse or rat genome. Firstly, a primer set to Na_V_1.7 was designed to recognized both mouse and rat isoforms. Only the rat Na_V_1.7 PCR product contains an *Eco*RI restriction site, producing two cleavage fragments of 344 bp and 312 bp, whereas the mouse PCR product (656 bp amplicon) does not harbor the restriction site. As shown in Fig 4B, the PCR product amplified using the Na_V_1.7 primer set was not digested by *Eco*RI, whereas control PCR product from rat DRG cDNA was cleaved by *Eco*RI in the same experiment, suggesting that mouse Na_V_1.7 is expressed in ND7/23. Secondly, to discriminate between the Na_V_1.9 isoforms, rat- and mouse-specific primer sets were respectively used in separate reactions. In similar fashion to the initial screen that used oligo-dT primed cDNA (Fig 2), we used a different mouse-specific primer set that resulted in a weak amplification of mouse Na_V_1.9 PCR product of 606 bp (Fig 4C). Whilst rat specific-primer set only amplified PCR product from the cDNA of rat DRG, there was no RT-PCR band from the cDNA of ND7/23. In the last experiment, Na_V_1.6 PCR amplicon of 1.9 kb size from ND7/23 cDNA that included the entire C-terminus of Na_V_1.6 channel was agarose gel-purified and sequenced to confirm fidelity of the amplicon DNA sequence with the mouse Na_V_1.6 sequence. Overall, these results indicate that only the mouse sodium channels Na_V_1.7 and Na_V_1.6 and Na_V_1.9 are expressed in ND7/23 cells.

## Materials and methods

### RNA isolation and cDNA synthesis

Total RNA from ND7/23 cells was extracted using Trizol reagent (Ambion, USA) according to the manufacturer’s protocol. Six-week old male Sprague Dawley rat (Orient Bio, Korea) dorsal root ganglia (DRG) cDNA was used as positive control templates to validate the specificity of some PCR primers. Fresh rat cadavers were used to dissect DRG. Animals were delivered on the day of experiment and sacrificed via CO_2_ inhalation for another study as approved by Seoul National University IACUC (SNU-160202-3-2, Dr. Seog Bae Oh). Tissue was collected in RNAlater solution (Ambion, USA), washed using Diethylpyrocarbonate (DEPC)-PBS and homogenized in Trizol reagent and RNA extracted.

mRNA was reverse transcribed (RT) in 6μl reaction containing M-MLV reverse transcriptase (200 units, Promega, USA), 50mM Tris-HCl (pH 8.3), 75mM KCl, 3mM MgCl_2_, 10mM dithiothreitol, dNTPs each at 0.5mM and RNase inhibitor (25 units, Enzynomics, Korea). Complementary DNA (cDNA) was synthesized from 500ng of total RNA, freshly isolated, with reverse transcriptase primed with (40pmole) oligo-dT or gene-specific primers. The cDNA was stored at -20°C until use.

### PCR

Polymerase chain reaction (PCR) was performed using 0.5 Units *Ex Taq* polymerase (Takara, Japan), 1μl of cDNA template from RT reaction, forward and reverse primers each at 0.8μM, dNTP each at 200μM, in 1x *Ex Taq* Buffer (2mM Mg^2+^) in 20μl reaction. Modified touchdown PCR protocol was used denaturing at 95°C for 30s where the annealing (primer-specific starting temperature of 71–65°C for 20s) was progressively decreased at a rate of 0.4°C per cycle for 20–25 cycles followed by 10 cycles of fixed annealing temperature in Biorad thermocycler (USA). Oligonucleotide primers (Macrogen, Korea) were designed to amplify mouse genes and rat *Scn11a* (Na_V_1.9) (S1 Table). To confirm amplicon sizes and the specificity of PCR, the mouse primer sets Na_V_1.6, Na_V_1.7, Na_V_1.8, Na_V_1.9-a, Na_V_1.9-b, β1, β2, β3, β4 were tested to confirm expected amplifications in control PCR using respective mouse cDNA templates.

To distinguish the mouse (neuroblastoma) or rat (embryonic DRG) origin of the sodium channel transcripts, the presence or absence of the restriction enzyme (*Eco*RI) site was used to analyze the Na_V_1.7 and Na_V_1.8 amplicons. To discriminate Na_V_1.9, individual mouse- and rat-specific primer sets were designed (S1 Table). PCR products and fragments resulting from enzyme digestion were size-fractionated on 2% agarose gel, stained with SafeView (CMI BIO, Korea) and visualized under UV gel doc.

### Quantitative real-time PCR

Gene-specific mRNA analyses were performed using the SYBR Premix *Ex Taq* (Takara, Japan) and the 7500 real-time PCR detection system (Biorad, USA). cDNAs of Na_V_1.6, Na_V_1.7 and Na_V_1.9 channels were amplified in 20μl PCR reactions using 1μl of RT reaction. Reactions with appropriate melting curve were selected for analysis and all primer sets were analyzed in triplicate. The β-actin RNA level was used as an internal control to normalize the values for transcript abundance of Na_V_1.6, Na_V_1.7 and Na_V_1.9 channels genes (S2 Table). Relative quantification (RQ: 2^−ΔCT^x100) was calculated using ΔC_T_ = C_T_ of target gene–C_T_ of reference gene, as previously described [23]. We performed three independent qRT-PCR experiments with each data point averaged from triplicate analysis of each experiment.

### Cell culture

ND7/23 cells (ECACC General Cell Collection #92090903) were purchased from Sigma-Aldrich (St. Louis, USA); cells were cultured in DMEM with 10% fetal bovine serum (PAN Biotech, Germany), supplemented with 2mM L-glutamate, 10% penicillin/streptomycin (Welgene, Korea). Cells were grown in humidified incubator (37°C, 5% CO_2,_). Growing cells were maintained for less than 10 passages by re-seeding at a lower density when 70~80% confluency was reached, and detached with 2.5mM EDTA in nominally Ca^2+^-free PBS. For patch-clamp recordings, one or two drops of detached cells were seeded onto 35mm culture dishes to promote single cell growth and used within 24-48hr. For extraction of RNA, ND7/23 cells were grown to 70~80% confluence in 100mm culture dishes.

### Whole-cell patch-clamp electrophysiology

Whole-cell patch-clamp recordings were performed at room temperature (20–25°C) using an EPC10 USB amplifier (HEKA Elektronik, Germany) that was controlled using Patchmaster software (v.2x73.5, HEKA). Fire-polished patch electrodes were prepared from borosilicate glass (1.5mm o.d., Sutter Instruments, USA) using a micropipette puller (Sutter model P97) with capillary resistance of 1–2.5 MΩ and used without electrode coating. The intracellular solution comprised of (in mM) 100 CsCl, 30 CsF, 10 EGTA, 1 CaCl_2_, 8 NaCl, 1 MgCl_2_, 0.4 Na_2_GTP, 4 MgATP, 10 HEPES and adjusted to 270–280 mOsm. Single cells (unattached to neighboring cells) were identified under microscope and were continuously bathed in the extracellular solution containing 140 NaCl, 2 CaCl_2_, 2 MgCl_2_, 10 HEPES 10 glucose, pH7.3 adjusted with NaOH. All chemicals were from Sigma-Aldrich. Offset potential was zeroed prior to each patch and capacitive transients were cancelled using computer-controlled circuitry of the EPC amplifier. Series resistance errors (typically less than 5 mV) were compensated with 75–90% series resistance compensations. Leak currents were linearly canceled by P/4 subtraction. Membrane current signals were filtered at 5kHz and sampled at 20kHz. Families of sodium currents were elicited by 100ms square test pulse ranging from –80 to +50mV in 10mV steps preceded by 150ms prepulse to –120mV from a holding potential of –70mV. Steady state inactivation used test pulse to 0mV, preceded by 500 ms long prepulses from –100 to +20mV. The holding potential was –120mV.

TTX (Alomone Labs, Israel) diluted in external bath solution was perfused directly onto cells through gravity-fed flow micropipettes positioned above the cell. Patched cells were held at –70mV, prepulsed to –120mV for 25 ms, followed by test pulse to 0mV for 30 ms. This protocol was repeated every 30 s during the experiment as long as the ‘gigaseal’ was retained (routinely 20 min in duration).

### Data analysis

The whole-cell patch-clamp experiments were analyzed using IgorPro software (v.6.37 Wavemetrics Inc., USA). Conductance-voltage (*G*–*V*) relationships were calculated from the current–voltage (*I–V*) relationship according to the extended Ohm’s law: *G* = *I*_Na_/(*V*–*E*_Na_), where *I*_Na_ is the peak Na^+^ current measured at potential *V*, and *E*_Na_ is the calculated Nernst equilibrium potential determined from the *V*-intercept of the linear fit of the *I*-*V* curve between 0 and +50 mV. The normalized *G–V* relationships and inactivation curves were fit with a Boltzmann distribution: 1/(1+exp[(*V–V*_1/2_)/*k*]), where *V*_1/2_ is the voltage at which half-activation or half-inactivation occurred, and *k* is the slope factor. Statistical results are reported as means±SD unless indicated.

Statistical comparisons were done using Microsoft Excel by one-way analysis of variance (α, 0.01) followed by post-hoc Student’s *t*-test with Bonferroni correction and *p* <0.0033 were considered significant.

## Discussion

The aim of the present study was to determine the molecular profile of endogenous voltage-gated sodium channels (VGSCs) that contribute to the sodium inward currents in ND7/23 cells. Our results complement prior studies of others that reported endogenous TTX-sensitive (TTX-S) sodium currents in these cells [9, 18, 21]. We found major expression of TTX-TTX-S Na_V_1.6 and Na_V_1.7 at similar levels and a minor expression of TTX-resistant (TTX-R) Na_V_1.9 and no expression of Na_V_1.8 in ND7/23 cells. In addition, our results also showed that only the mouse forms of Na_V_1.6, Na_V_1.7 and Na_V_1.9 channels were expressed in ND7/23 cells that was originally generated as a hybridoma of rat embryonic DRG and mouse N18Tg2 neuroblastoma cell-line [15].

Although several studies have used molecular techniques to identify the presence of mRNA for VGSC family in ND7/23 cells, the variety and levels of sodium channel expression have been mixed. The study by Leffler *et al*. (2010), in which primers were designed to recognize both the mouse and rat forms of Na_V_1.1, Na_V_1.2, Na_V_1.3, Na_V_1.4, Na_V_1.6 and Na_V_1.7, suggested that ND7/23 cells have no expression of Na_V_1.6 –only Na_V_1.2, Na_V_1.3 and Na_V_1.7 transcripts were reported in their study [21]. However, a recent study that examined dysregulation of sodium channels in post-herpetic neuralgia using ND7/23–rNav1.8 stable cell-line, indicated that Na_V_1.6 isoform was indeed expressed in these cells [24]. They showed by end-point RT-PCR that low levels of Na_V_1.3, and robust and similar levels of endogenous Na_V_1.6 and Na_V_1.7 were expressed along with the exogenous rNa_V_1.8 in the ND7/23–rNa_V_1.8 cells. The results of our experiments also confirmed that the expression of Na_V_1.6 transcript in ND7/23 cells by direct DNA sequencing of the amplified RT-PCR product. Furthermore, the Na_V_1.6 levels were virtually similar to that of Na_V_1.7 transcripts (Fig 3B). These results are in good agreement with the study by Kennedy *et al*. (2013) [24], and much different with the study by Rogers *et al*. (2016) that implied 1:3 ratio of Na_V_1.6: Na_V_1.7 based on the sodium current measurements using ‘specific’ blockers to each sodium channel [25]. Approximately 60~65% of TTX-S current in ND7/23 cell were blocked by the tarantula spider toxin ProToxin-II which is known to specifically block Na_V_1.7 and only ~20% of the current were blocked by Na_V_1.6 specific inhibitor, 4.9-anhydro-TTX. Although ProToxin-II has been used as specifically inhibitor of Na_V_1.7, it has effects on other sodium channel subtypes. At the 10nM concentration used to completely inhibit Na_V_1.7 channels, it would block approximately ~30% of Na_V_1.6 [26], suggesting that the ProToxin-II-inhibitable current in ND7/23 cell as reported by Rogers *et al*. (2016) may be an overestimation. Although there is typically a good correlation of the transcript with the cell protein levels, it cannot be rule out that in ND7/23 cells sodium channel protein expression may not correlate directly with the transcript levels.

The main goal of our study was to carefully examine the expression of the TTX-R sodium channel isoforms, Na_V_1.8 and Na_V_1.9, as these have been refractory to expression and study in other heterologous expression systems. Our ultimate goal is to characterize the regulation and function of these channels in ND7/23 cells. In addition to oligo-dT primed cDNA, our study also employed specific primer complementary to Na_V_1.8 coding region to generate the first-strand cDNA. Both reactions did not reveal any Na_V_1.8-specific PCR amplifications, suggesting that Na_V_1.8 channels are not expressed. Also absent were Na_V_1.5 (and Na_V_1.4) channels. However, Na_V_1.9 transcripts were indeed expressed, *albeit* at ~270-fold lower level compared to Na_V_1.7 transcripts (Fig 3B). Overall however, the lack of TTX-R sodium currents by whole-cell patch-clamp (Fig 1A) and no or low expression of Na_V_1.8 and Na_V_1.9 transcripts suggest that ND7/23 cells are essentially null for TTX-R sodium currents. Other sodium channel isoforms Na_V_1.1, Na_V_1.2, Na_V_1.3 whose genes are also found at the same chromosomal locus as those of Na_V_1.6 and Na_V_1.7 that are robustly expressed in ND7/23 cells, showed much lower levels of expression.

Apparently, ND7/23 cells are more amenable to express TTX-R sodium channels Na_V_1.8 [9] and Na_V_1.9 [17] whilst HEK and CHO cells may be constrained despite being suitable heterologous cell models to express diverse array of recombinant ion channels, including many other voltage-gated sodium channels [27–29]. The reasons for this are not clear. Consistent expression of human Na_V_1.9 in ND7/23 cells was attained with a transposon-based cDNA expression system [17] and low temperature incubation after transfection in similar strategy to the temperature-sensitive CFTR mutant protein delivery to plasmalemma [30]. Possibly, the neuron-like *milieu* of ND7/23 cells unlike the epithelia-like HEK and CHO cells may provide intracellular conditions and/or accessory protein factors that are conducive to the expression of recombinant TTX-R sodium channels [17]. Recently, stable expression of human and rodent Na_V_1.9 channels with sufficient current density were produced in HEK293 cells following stable co-expression of auxiliary β1 and β2 subunits along with 28°C incubation and the addition of GTP-γ-S in the intracellular solution to maximize recorded currents for automated patch clamp-based pharmaceutical discovery screening efforts [14]. Indeed, our results presented in Fig 2C also show the expression of only β1 and β3 subunits in ND7/23 cells. Together with previously reported characterization of human Na_V_1.9 that did not cotransfect beta subunit cDNA [17] suggest that β2 (or β4) subunits that associate through covalent interactions with some TTX-S sodium channel α-subunits [1, 3] may not be required for expression of Na_V_1.9 channels in these cells. Indeed, ability to transiently express recombinant TTX-R sodium channels in ND7/23 cell (with defined background of sodium currents) should permit unhindered studies of Na_V_1.8 and Na_V_1.9 channels, as well as characterization of their channelopathy-associated mutations.

We performed comprehensive screening of the background sodium channel subtypes present in ND7/23 cells. Our results show that sodium currents resistant to 100nM TTX are not observed in ND7/23 cells, specifically no Na_V_1.8 transcripts and very low transcript levels of Na_V_1.9. We also show that major sodium currents of ND7/23 cells are TTX-S Na_V_1.7, and Na_V_1.6 and that there are minor expression of Na_V_1.1, Na_V_1.2, Na_V_1.3 channels. In addition, β1 and β3, but not the β2 and β4 subunits that covalently associate with sodium channel α-subunits were expressed in ND7/23 cells. Evidently, nanomolar TTX block is needed to study transfected sodium currents in these cells. Despite this limitation, our results agree with accumulating data that ND7/23 cells are a good cell model to perform detailed study of the function and regulation of TTX-R sodium channels of peripheral nervous system that are linked to human genetic pain disorders and that are garnering attention as important potential therapeutics-development targets in inflammatory and chronic pain signaling.

## Supporting information

S1 Fig Additional RT-PCR analyses comparing expression of some TTX-S channel transcripts in ND7/23 cells. (A, B) Mouse Na_V_1.6 and Na_V_1.7 showed similar levels of amplification that appeared to be consistently greater than the amplifications of mouse Na_V_1.1 or Na_V_1.2, or Na_V_1.3 bands. Presence (+ symbol) or absence (–symbol) of oligo-dT primed ND7/23 cDNA, M- 1kb DNA marker. (C, *Left panel)* Control PCR of mouse beta 2 and beta 4 primers (Fig 4C) using cDNAs of mouse β2 and β4 as templates. The reactions produced expected size amplicons of 648 bp and 687 bp, respectively, suggesting that lack of signal in Fig 4A is not failure to recognize mouse sequences. (C, *Right panel)*. Control PCR of mouse Na_V_1.8 and Na_V_1.9 primers used in Figs 2A and 4A using dH_2_O (NC), ND7/23 total RNA (RNA), cDNA of mouse Na_V_1.8 or Na_V_1.9 (PC). Lack of signals for Na_V_1.8 in Figs 2A and 4A is not failure to recognize mouse sequences.
(TIF)

S2 Fig Original uncropped gel images. (A) Original non-cropped image of Fig 2A. (B) Original non-cropped image of Fig 4A.
(TIF)

S1 Table Oligonucleotide primers used in RT-PCR. (DOCX)

S2 Table Oligonucleotide primers used in quantitative RT-PCR. (DOCX)
