# Supplementary figures and images for: NaV1.6 and NaV1.7 channels are major endogenous voltage-gated sodium channels in ND7/23 cells
Source: PLoS One. 2019 Aug 16;14(8):e0221156. doi: 10.1371/journal.pone.0221156 (PMC6697327; doi:10.1371/journal.pone.0221156)

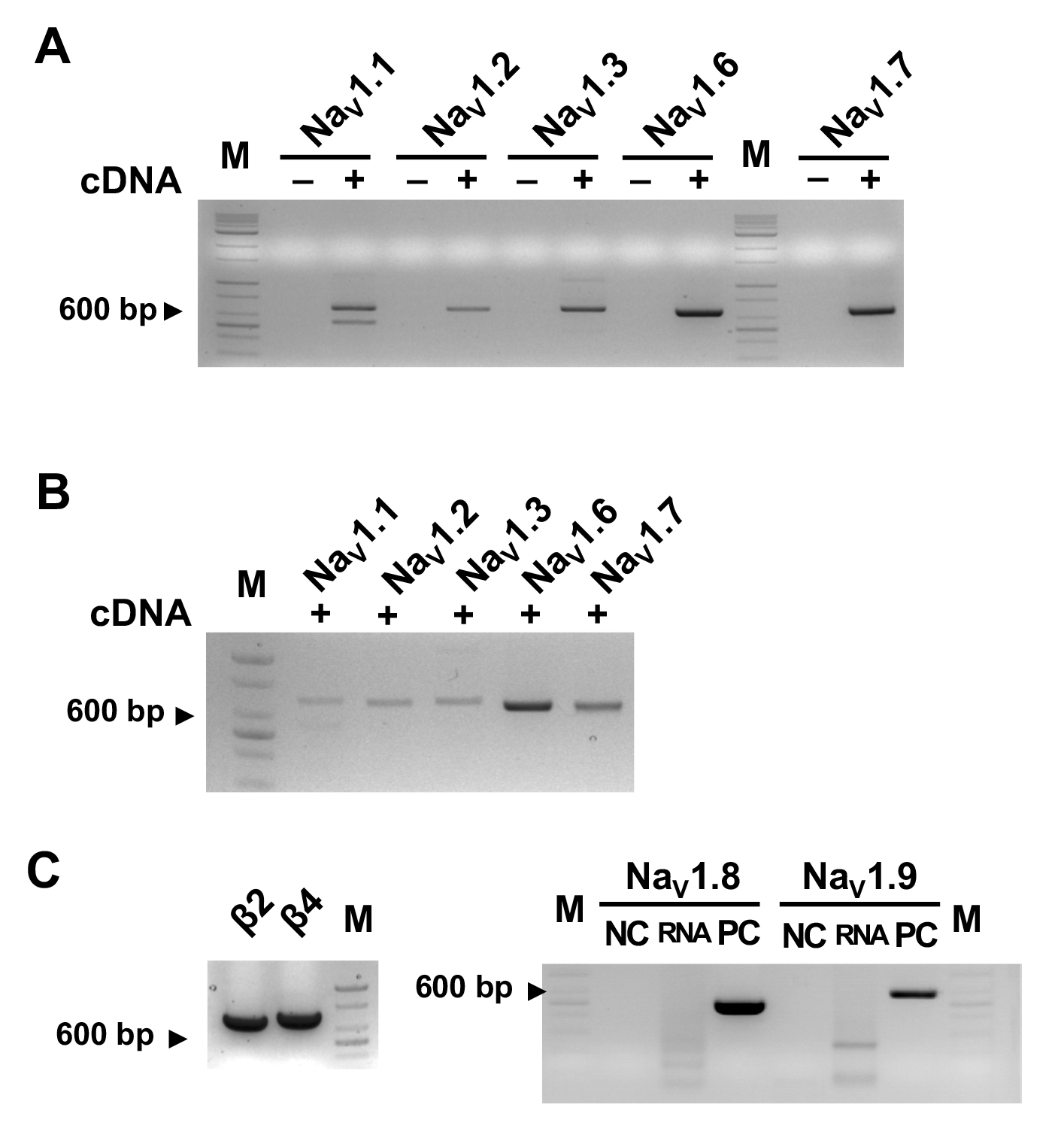

Supplement: S1 Fig — (A, B) Mouse NaV1.6 and NaV1.7 showed similar levels of amplification that appeared to be consistently greater than the amplifications of mouse NaV1.1 or NaV1.2, or NaV1.3 bands. Presence (+ symbol) or absence (–symbol) of oligo-dT primed ND7/23 cDNA, M- 1kb DNA marker. (C, Left panel) Control PCR of mouse beta 2 and beta 4 primers (Fig 4C) using cDNAs of mouse β2 and β4 as templates. The reactions produced expected size amplicons of 648 bp and 687 bp, respectively, suggesting that lack of signal in Fig 4A is not failure to recognize mouse sequences. (C, Right panel). Control PCR of mouse NaV1.8 and NaV1.9 primers used in Figs 2A and 4A using dH2O (NC), ND7/23 total RNA (RNA), cDNA of mouse NaV1.8 or NaV1.9 (PC). Lack of signals for NaV1.8 in Figs 2A and 4A is not failure to recognize mouse sequences. (TIF) [file pone.0221156.s001.tif]

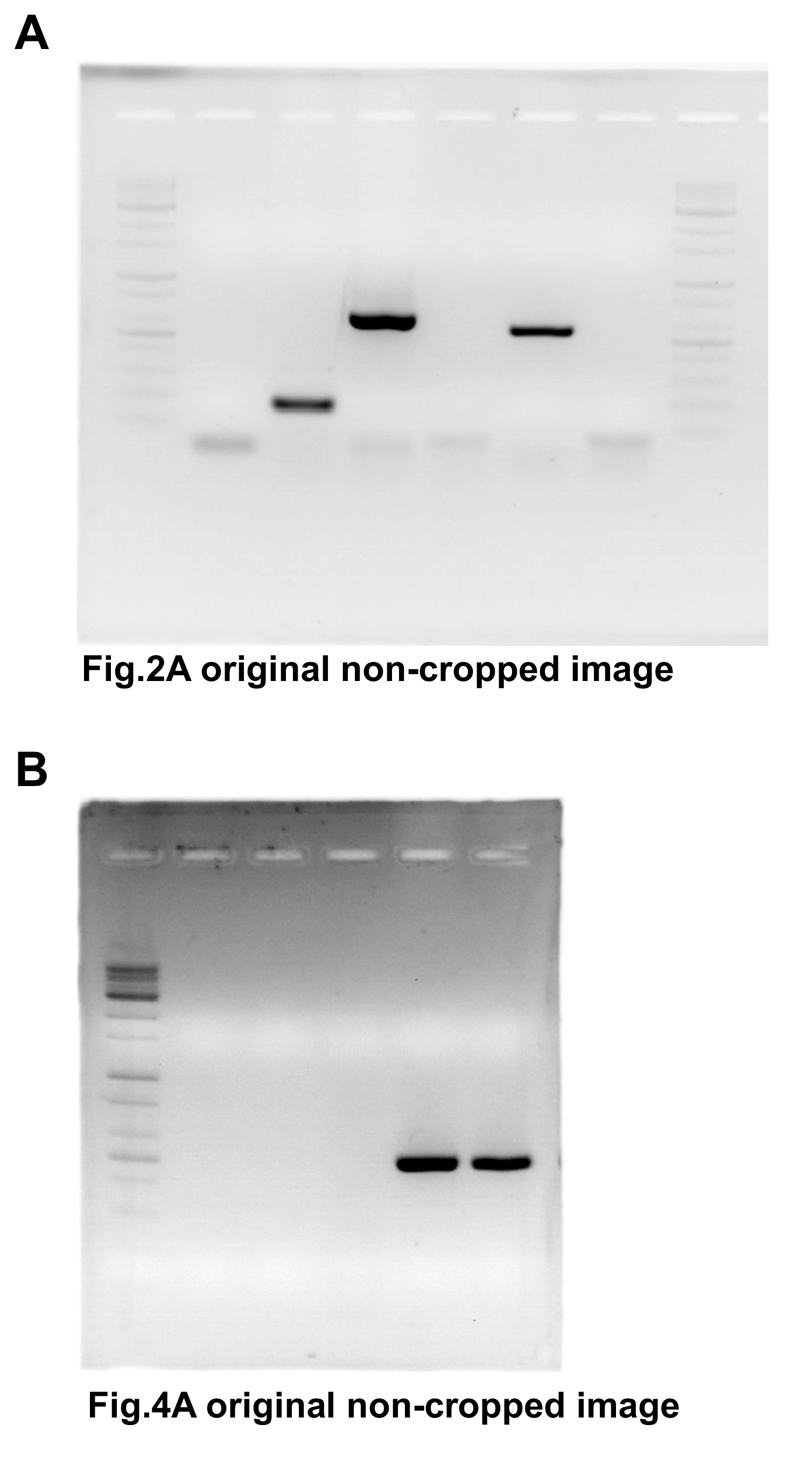

Supplement: S2 Fig — (A) Original non-cropped image of Fig 2A. (B) Original non-cropped image of Fig 4A. (TIF) [file pone.0221156.s002.tif]
