# Supplementary material for: NaV1.6 and NaV1.7 channels are major endogenous voltage-gated sodium channels in ND7/23 cells
Source: PLoS One. 2019 Aug 16;14(8):e0221156. doi: 10.1371/journal.pone.0221156 (PMC6697327; doi:10.1371/journal.pone.0221156)
Supplement: S1 Table — (DOCX) [file pone.0221156.s003.docx]

| **S1 Table. Oligonucleotide primers used in RT-PCR^#^** | | |  |
| --- | --- | --- | --- |
|  |  |  |  |
| **Name (Gene)** | **F /R** | **Primer sequence 5'-3'** | **expected**  **length (bp)** |
| Na_V_1.1 | F | CCGTGTTCGAGTTTGACTGGAA | 683 |
| *(Scn1a)* | R | GCGAAACCCCTTCCTCCTG |  |
| Na_V_1.2 | F | GGAATGGTACTGCCTTCAATAGG | 674 |
| *(Scn2a)* | R | AGATTTCCGCACAGCATCTTC |  |
| Na_V_1.3 | F | TGGCACAATGGACTCAAATGG | 681 |
| *(Scn3a)* | R | AACCTGTCTCCTTCGGGTC |  |
| Na_V_1.4* | F | GCCCATGAATGACACCAACA | 368 |
| *(Scn4a)* | R | GCTGGAAAAGGTTCTCCCAG |  |
| Na_V_1.5 | F | CAATGGTTCCGTGGAGGC | 589 |
| *(Scn5a)* | R | TGTCCCTGAAGACAGTCGTTT |  |
| Na_V_1.6 | F | CGAGAGCTATCTGGAGAACGG | 662 |
| *(Scn8a)* | R | CCTTCTCCTCCCCTTCAGAG |  |
| Na_V_1.7^*^ | F | GAAACCTCCAGGCTGAGCTC | 656 |
| *(Scn9a)* | R | GGGTCATTCAGCATGTCCTCAG |  |
| Na_V_1.8^*^ | F | CAGCACACACCGGACATTCA | 521 |
| *(Scn10a)* | R | CTCAGGCAGAAGACTGTGAGG |  |
| Na_V_1.9-a | F | GTGATTCTAGAGAACTTCAACACAGC | 564 |
| *(Scn11a)* | R | GTGCTCGAGTCAGTCACAATGAACCTTGATCTTGG |  |
| Na_V_1.9-b | F | GAAGATGCATTAATGGAACAGACATAAAT | 606 |
|  | R | AGACTATGTTGAGAATATCAAAGATTTTCTT |  |
| rNa_V_1.9 | F | GTGATTCTAGAGAACTTCAACACAGC | 564 |
|  | R | GTGCTCGAGTCAGTCATTGTGAACCTTGACCTTG |  |
| Gapdh | F | GTCTTCACCACCATGGAGA | 170 |
|  | R | AAGCAGTTGGTGGTGCAG |  |
| β1 | F | AAAAGCTTAACATGGGGACGCTGCTGGCTCTC | 657 |
| *(Scn1b)* | R | AAACTCGAGCTATTCAGCCACCTGGACGCCTGT |  |
| β2 | F | AAAAGCTTACCATGCACAGGGATGCCTGGCTAC | 648 |
| *(Scn2b)* | R | AAACTCGAGCTACTTGGTGCCATCTTCCGCGTTG |  |
| β3 | F | AAAAGCTTAACATGCCTGCCTTCAACAGATTGCTT | 648 |
| *(Scn3b)* | R | AAACTCGAGCTATTCCTCCACGGGTACCACAGAG |  |
| β4 | F | AAAAGCTTACCATGTCCCGGGCAGGGAACCGAG | 687 |
| *(Scn4b)* | R | AAACTCGAGCTACACTTTTGTGGGTGGCTTCTCTTC |  |
| Na_V_1.6-seq | F | CATGGGCGTTAACCTGTTTGC | 1924 |
|  | R | AAACTCGAGCTAGCACTTGGACTCCCTGAC |  |
| ^#^ All primer sets span at least one exon-intron junction, except for rNa_V_1.9  * Primers anneal to mouse and rat sequences. | | |  |
