# Supplementary material for: NaV1.6 and NaV1.7 channels are major endogenous voltage-gated sodium channels in ND7/23 cells
Source: PLoS One. 2019 Aug 16;14(8):e0221156. doi: 10.1371/journal.pone.0221156 (PMC6697327; doi:10.1371/journal.pone.0221156)
Supplement: S2 Table — (DOCX) [file pone.0221156.s004.docx]

| **S2 Table. Oligonucleotide primers used in quantitative RT-PCR** | | |  |
| --- | --- | --- | --- |
|  |  |  |  |
| **Target** | **F /R** | **Primer sequence 5'-3'** | **product**  **length (bp)** |
| Na_V_1.6 | F | ATGGTGAGCGGAGATCGAA | 161 |
|  | R | GTGGTCGTGATAGGCTCGTA |  |
| Na_V_1.7 | F | TCCTTTATTCATAATCCCAGCCTCAC | 134 |
|  | R | GATCGGTTCCGTCTCTCTTTGC |  |
| Na_V_1.9 | F | GGGTCCTGTGGTGGAATCTT | 112 |
|  | R | TCGAATATCAGTGCTCCGCT |  |
| β-actin | F | GACAGGATGCAGAAGGAGATTACTG | 98 |
|  | R | CCACCGATCCACACAGAGTACTT |  |
| Pmca4 | F | CATCTCACTAGCCTACTCTGT | 117 |
|  | R | GTGCCTGTCTTATCGGAGCAA |  |
